# 1,3-Dinitro­soimidazolidine

**DOI:** 10.1107/S1600536812030796

**Published:** 2012-07-14

**Authors:** Augusto Rivera, Diego Quiroga, Jaime Ríos-Motta, Michal Dušek, Karla Fejfarová

**Affiliations:** a Universidad Nacional de Colombia, Sede Bogotá, Facultad de Ciencias, Departamento de Química, Cra 30 No. 45-03, Bogotá, Código Postal 111321, Colombia; b Institute of Physics ASCR, v.v.i., Na Slovance 2, 182 21 Praha 8, Czech Republic

## Abstract

The title compound, C_3_H_6_N_4_O_2_, exhibits partial disorder with the refined occupancy ratios of the two components being 0.582 (5):0.418 (5). In the major component, the nitroso groups have a relative *syn* spatial arrangement [O=N⋯N=O pseudo-torsion angle = 1.1 (4)°], whereas the other component has an *anti* disposition [177.6 (1)°]. The N—N=O moieties are almost coplanar with a dihedral angle of 5.3 (3)°, while in the minor occupied set of atoms, this angle is 8 (1)°. In both components, the imidazolidine ring adopts a twisted conformation on the C—C bond and the crystal structure shows the strain of this ring according to the N—CH_2_—CH_2_—N torsion angles [25.9 (5) and −23.8 (7)°]. In the crystal, molecules are linked by weak C—H⋯O hydrogen bonds.

## Related literature

For a related structure, see: Rivera *et al.* (2011 ▶). For the synthesis of the title compound, see: Rivera *et al.* (1997 ▶). For ring conformations, see Cremer & Pople (1975 ▶). For chemical background on the synthesis and uses of *N*-nitroso amines, see: Di Salvo *et al.* (2008 ▶).
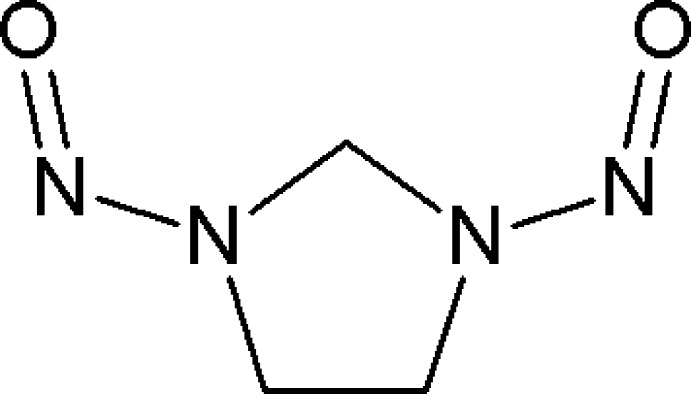


## Experimental

### 

#### Crystal data

C_3_H_6_N_4_O_2_
*M* *_r_* = 130.1
Orthorhombic, 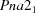

*a* = 9.5154 (2) Å
*b* = 5.4338 (1) Å
*c* = 10.7104 (2) Å
*V* = 553.78 (2) Å^3^
*Z* = 4
Cu *K*α radiation
μ = 1.14 mm^−1^
*T* = 120 K
0.39 × 0.20 × 0.14 mm

#### Data collection

Agilent Xcalibur diffractometer with an Atlas (Gemini Ultra Cu) detector
Absorption correction: multi-scan (*CrysAlis PRO*; Agilent, 2010 ▶) *T*_min_ = 0.636, *T*_max_ = 1
5160 measured reflections
522 independent reflections
514 reflections with *I* > 3σ(*I*)
*R*_int_ = 0.031

#### Refinement

*R*[*F* ^2^ > 2σ(*F* ^2^)] = 0.039
*wR*(*F* ^2^) = 0.126
*S* = 2.86
522 reflections
87 parameters
H-atom parameters constrained
Δρ_max_ = 0.12 e Å^−3^
Δρ_min_ = −0.19 e Å^−3^

### 

Data collection: *CrysAlis PRO* (Agilent, 2010 ▶); cell refinement: *CrysAlis PRO*; data reduction: *CrysAlis PRO*; program(s) used to solve structure: *SIR2002* (Burla *et al.*, 2003 ▶); program(s) used to refine structure: *JANA2006* (Petříček *et al.*, 2006 ▶); molecular graphics: *DIAMOND* (Brandenburg & Putz, 2005 ▶); software used to prepare material for publication: *JANA2006*.

## Supplementary Material

Crystal structure: contains datablock(s) global, I. DOI: 10.1107/S1600536812030796/bt5956sup1.cif

Structure factors: contains datablock(s) I. DOI: 10.1107/S1600536812030796/bt5956Isup2.hkl

Supplementary material file. DOI: 10.1107/S1600536812030796/bt5956Isup3.cml

Additional supplementary materials:  crystallographic information; 3D view; checkCIF report

## Figures and Tables

**Table 1:** Hydrogen-bond geometry (Å, °)

*D*—H⋯*A*	*D*—H	H⋯*A*	*D*⋯*A*	*D*—H⋯*A*
C3*y*—H3*ya*⋯O1*y* ^i^	0.96	1.85	2.681 (12)	143

Symmetry code: (i) 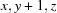.

**Fig. 1.**
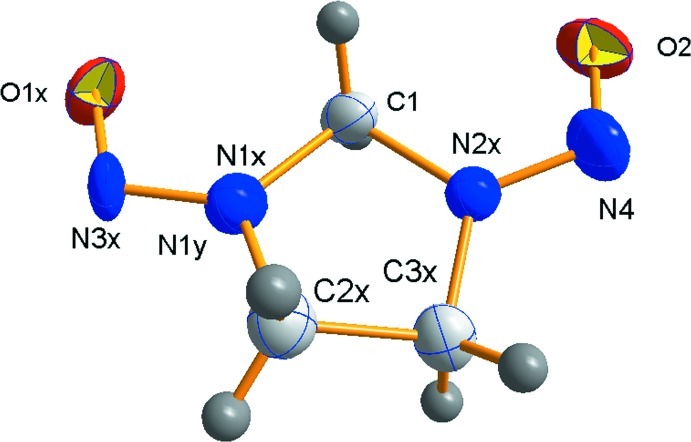
A view of the title compound with the numbering scheme. Only the major disordered component is shown. Displacement ellipsoids are drawn at the 50% probability level.

**Fig. 2.**
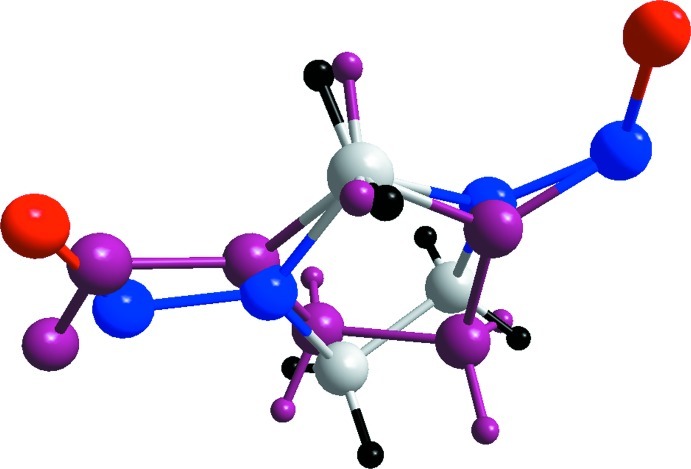
Overlapped structures of disordered components, showing structural differences.

**Fig. 3.**
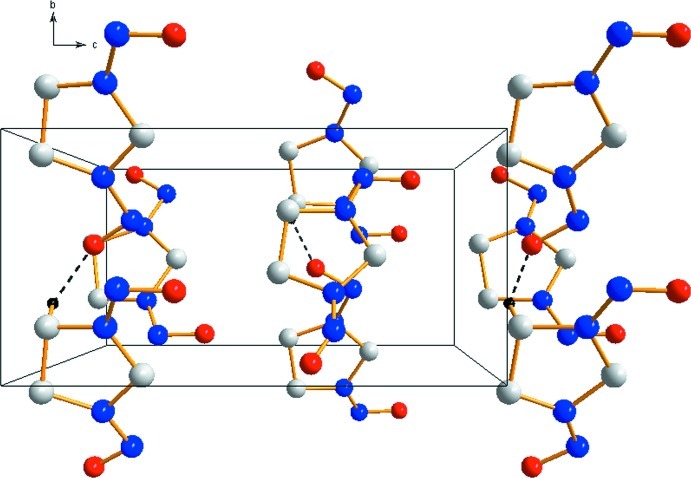
The packing of the title compound, viewed along the a axis. The dashed lines indicate the hydrogen bonds. H atoms not involved in hydrogen bonding have been omitted for clarity.

## supplementary crystallographic information

### Comment

*N*-nitrosamines are interesting molecules due their strong carcinogenic and mutagenic properties and their utility as synthetic intermediates for the preparation of various *N,N*-bonded functionalities (Di Salvo *et al.*, 2008). Our group has previously explored the reaction of nitrous acid with cyclic aminals which actually are tertiary amines (Rivera *et al.*, 1997, 2011). Earlier we reported the synthesis and complete characterization by NMR of the title compound 1,3-dinitrosoimidazolidine, obtained by the nitrosation reaction of the cyclic aminal 1,3,6,8-tetraazatricyclo[4.4.1.1.^3,8^]dodecane (Rivera *et al.*, 1997). NMR experiments of this compound evidenced the existence of a mixture of three isomers: *syn-cis*, *anti*, and *cis-trans* with a ratio of 31:50:19 respectively (Rivera, *et al.* 1997). However, a recently investigation of (3aRS,7aRS)-1,3-dinitrosooctahydro-1*H*-benzimidazole, we found that the nitroso groups of this analogous X-ray crystal structure are on a *syn-cis* disposition (Rivera, *et al.* 2011). This result suggests that the orientation of the nitroso groups on the imidazolidine ring is largely influenced by their molecular skeletons. To identify the orientation of nitroso groups, we synthesized the title compound and investigated its crystal structure.

X-Ray analysis confirms the molecular structure and atom connectivity as illustrated in Fig. 1. The bond lengths N—C1 and N—NO are normal and comparable to the corresponding values observed in the related structure (Rivera, *et al.* 2011). The title compound are disordered over two sets of sites [site occupancies = 0.588 (6) and 0.412 (6)]. In both components, the N—N═O moieties are almost coplanar showing dihedral angles of 5.277 (340)° for the major component and 7.81 (97)° for the minor. The nitroso substituents in the major component are on a *syn* spatial arrangement as can be seen from O1x═N3x···N4═O2 pseudo torsion angle of = 1.119 (410)°, whereas the other component have an *anti* disposition [pseudo torsion angle O1y═N3y···N4═O2 = 177.662 (129)°] (Figure 2). Both imidazole ring system are twisted on CH_2_CH_2_ fragment as seen in the puckering parameters Q(2) = 0.255 (5) Å and φ2 = 122.7 (12)° for major component and Q(2) = 0.236 (8) Å and φ2 = 90.9 (16)° for minor component (Cremer & Pople, 1975). The crystal structure shows the strain of this ring according to the NCH_2_CH_2_N torsion angles [N1xC2xC3xN2x = 25.874 (534)° and N1yC2yC3yN2y = -23.808 (735)°].

The crystal packing displays a weak intermolecular C—H···O [C···O = 2.681 (12) Å] non-conventional hydrogen bonding interactions between oxygen atoms in the nitroso moiety and hydrogen atoms in methylene carbons of the heterocyclic ring (Figure 3).

### Experimental

For the originally reported synthesis, see: Rivera *et al.* (1997). Single crystals of the title compound were obtained by recrystallization from EtOH solution (m.p 318 K).

### Refinement

All hydrogen atoms were positioned geometrically and treated as riding on their parent atoms. The isotropic atomic displacement parameters of hydrogen atoms were set to 1.2×*U*_eq_ of the parent atom.

The molecule is disordered over two positions with occupancies 0.588 (6):0.412 (6). Selected atoms of both components were refined isotropically as anisotropic refinement lead to unreasonable ADPs.

As the structure contains only light atoms, the Friedel-pairs were merged and the Flack parameter has not been determined.

### Crystal data

**Table:**

C_3_H_6_N_4_O_2_	*F*(000) = 272
*M**_r_* = 130.1	*D*_x_ = 1.56 Mg m^−^^3^
Orthorhombic, *P**n**a*2_1_	Cu *K*α radiation, λ = 1.5418 Å
Hall symbol: P 2c -2n	Cell parameters from 4445 reflections
*a* = 9.5154 (2) Å	θ = 4.1–66.8°
*b* = 5.4338 (1) Å	µ = 1.14 mm^−^^1^
*c* = 10.7104 (2) Å	*T* = 120 K
*V* = 553.78 (2) Å^3^	Prism, colourless
*Z* = 4	0.39 × 0.20 × 0.14 mm

### Data collection

**Table:**

Agilent Xcalibur diffractometer with an Atlas (Gemini Ultra Cu) detector	522 independent reflections
Radiation source: Enhance Ultra (Cu) X-ray Source	514 reflections with *I* > 3σ(*I*)
Mirror monochromator	*R*_int_ = 0.031
Detector resolution: 10.3784 pixels mm^-1^	θ_max_ = 67.0°, θ_min_ = 9.2°
Rotation method data acquisition using ω scans	*h* = −11→11
Absorption correction: multi-scan (*CrysAlis PRO*; Agilent, 2010)	*k* = −6→6
*T*_min_ = 0.636, *T*_max_ = 1	*l* = −12→12
5160 measured reflections	

### Refinement

**Table:**

Refinement on *F*^2^	86 constraints
*R*[*F*^2^ > 2σ(*F*^2^)] = 0.039	H-atom parameters constrained
*wR*(*F*^2^) = 0.126	Weighting scheme based on measured s.u.'s *w* = 1/(σ^2^(*I*) + 0.0016*I*^2^)
*S* = 2.86	(Δ/σ)_max_ = 0.013
522 reflections	Δρ_max_ = 0.12 e Å^−^^3^
87 parameters	Δρ_min_ = −0.19 e Å^−^^3^
0 restraints	

### Special details

**Table:**

Experimental. CrysAlisPro (Agilent Technologies, 2010) Empirical absorption correction using spherical harmonics, implemented in SCALE3 ABSPACK scaling algorithm.
Refinement. The refinement was carried out against all reflections. The conventional *R*-factor is always based on *F*. The goodness of fit as well as the weighted *R*-factor are based on *F* and *F*^2^ for refinement carried out on *F* and *F*^2^, respectively. The threshold expression is used only for calculating *R*-factors *etc*. and it is not relevant to the choice of reflections for refinement.The program used for refinement, Jana2006, uses the weighting scheme based on the experimental expectations, see _refine_ls_weighting_details, that does not force *S* to be one. Therefore the values of *S* are usually larger than the ones from the *SHELX* program.

### Fractional atomic coordinates and isotropic or equivalent isotropic displacement parameters (Å^2^)

**Table:**

	*x*	*y*	*z*	*U*_iso_*/*U*_eq_	Occ. (<1)
O1x	0.3214 (4)	−0.3809 (8)	0.266995	0.0436 (13)	0.582 (5)
O2	0.0084 (2)	0.3676 (5)	0.3407 (6)	0.0527 (8)	
N1x	0.2304 (5)	−0.1122 (9)	0.1438 (8)	0.0333 (9)*	0.582 (5)
N1y	0.2125 (8)	−0.1576 (12)	0.1559 (9)	0.0333 (9)*	0.418 (5)
N2x	0.0781 (4)	0.1899 (8)	0.1754 (7)	0.0292 (8)*	0.582 (5)
N2y	0.1109 (6)	0.2145 (12)	0.1820 (9)	0.0292 (8)*	0.418 (5)
N3x	0.3056 (5)	−0.3111 (9)	0.1569 (10)	0.0342 (15)	0.582 (5)
N3y	0.2972 (18)	−0.338 (3)	0.2034 (16)	0.063 (5)*	0.418 (5)
O1y	0.3425 (9)	−0.4441 (19)	0.1083 (12)	0.081 (2)*	0.418 (5)
N4	0.0159 (3)	0.3748 (4)	0.2258 (6)	0.0469 (9)	
C1	0.1576 (2)	0.0075 (6)	0.2498 (6)	0.0348 (8)	
C2x	0.2145 (5)	0.0048 (10)	0.0225 (8)	0.0369 (11)*	0.582 (5)
C2y	0.1820 (8)	−0.0681 (14)	0.0312 (9)	0.0369 (11)*	0.418 (5)
C3x	0.0820 (5)	0.1482 (9)	0.0410 (8)	0.0338 (13)	0.582 (5)
C3y	0.1527 (8)	0.2091 (13)	0.0512 (10)	0.0338 (13)	0.418 (5)
H2xa	0.199648	−0.11884	−0.0401	0.0443*	0.582 (5)
H2xb	0.291244	0.116195	0.008783	0.0443*	0.582 (5)
H2ya	0.098714	−0.146931	0.000258	0.0443*	0.418 (5)
H2yb	0.263608	−0.087618	−0.020601	0.0443*	0.418 (5)
H3xa	0.089387	0.303307	−0.0015	0.0406*	0.582 (5)
H3xb	0.00295	0.049365	0.016793	0.0406*	0.582 (5)
H3ya	0.23834	0.300237	0.040787	0.0406*	0.418 (5)
H3yb	0.074938	0.258704	−0.000047	0.0406*	0.418 (5)
H1ax	0.225236	0.091148	0.301089	0.0417*	0.582 (5)
H1bx	0.093385	−0.107021	0.28725	0.0417*	0.582 (5)
H1ay	0.232011	0.055714	0.30525	0.0417*	0.418 (5)
H1by	0.079178	−0.068441	0.29095	0.0417*	0.418 (5)

### Atomic displacement parameters (Å^2^)

**Table:**

	*U*^11^	*U*^22^	*U*^33^	*U*^12^	*U*^13^	*U*^23^
O1x	0.0389 (19)	0.053 (2)	0.039 (3)	0.0103 (15)	−0.0008 (19)	0.0147 (19)
O2	0.0448 (12)	0.0675 (15)	0.0458 (16)	0.0025 (10)	0.0059 (10)	−0.0244 (11)
N3x	0.031 (2)	0.036 (2)	0.036 (3)	0.0200 (15)	0.008 (2)	0.002 (2)
N4	0.0526 (17)	0.0417 (14)	0.0463 (17)	0.0098 (11)	0.0081 (13)	−0.0109 (11)
C1	0.0288 (13)	0.0449 (14)	0.0306 (14)	0.0034 (9)	−0.0021 (11)	−0.0035 (11)
C3x	0.038 (2)	0.031 (2)	0.033 (2)	0.0003 (18)	0.007 (2)	0.0029 (16)
C3y	0.038 (2)	0.031 (2)	0.033 (2)	0.0003 (18)	0.007 (2)	0.0029 (16)

### Geometric parameters (Å, º)

**Table:**

O1x—N3x	1.248 (10)	N2y—C1	1.411 (9)
O2—N4	1.233 (9)	N2y—C3y	1.457 (14)
N1x—N3x	1.303 (7)	N3y—O1y	1.25 (2)
N1x—C1	1.481 (9)	C1—H1bx	0.96
N1x—C2x	1.454 (11)	C1—H1ay	0.96
N1y—N3y	1.366 (17)	C1—H1by	0.96
N1y—C1	1.445 (10)	C2x—C3x	1.495 (7)
N1y—C2y	1.451 (13)	C2x—H2xa	0.96
N2x—N4	1.285 (6)	C2x—H2xb	0.96
N2x—C1	1.480 (7)	C2y—C3y	1.547 (11)
N2x—C3x	1.458 (12)	C3x—H3xb	0.96
N2y—N4	1.340 (8)	C3y—H3yb	0.96
			
N3x—N1x—C1	122.6 (7)	N1y—C1—H1ay	109.4716
N3x—N1x—C2x	121.0 (7)	N1y—C1—H1by	109.4714
C1—N1x—C2x	116.4 (4)	N2x—C1—H1bx	109.4709
N3y—N1y—C1	113.5 (10)	N2y—C1—H1ay	109.4711
N3y—N1y—C2y	134.5 (10)	N2y—C1—H1by	109.4712
C1—N1y—C2y	111.1 (6)	N1x—C2x—C3x	101.4 (6)
N4—N2x—C1	122.2 (7)	N1x—C2x—H2xa	109.4711
N4—N2x—C3x	123.2 (5)	N1x—C2x—H2xb	109.4713
C1—N2x—C3x	114.5 (4)	C3x—C2x—H2xa	109.4718
N4—N2y—C1	123.4 (8)	C3x—C2x—H2xb	109.4715
N4—N2y—C3y	122.3 (7)	H2xa—C2x—H2xb	116.4713
C1—N2y—C3y	113.1 (6)	N1y—C2y—C3y	103.6 (7)
O1x—N3x—N1x	114.8 (8)	N2x—C3x—C2x	103.4 (6)
N1y—N3y—O1y	103.4 (13)	N2x—C3x—H3xb	109.4711
O2—N4—N2x	114.9 (5)	C2x—C3x—H3xb	109.4711
O2—N4—N2y	111.6 (5)	N2y—C3y—C2y	101.7 (7)
N1x—C1—N2x	96.9 (5)	N2y—C3y—H3yb	109.4711
N1x—C1—H1bx	109.4713	C2y—C3y—H3yb	109.4709
N1y—C1—N2y	104.5 (7)		

### Hydrogen-bond geometry (Å, º)

**Table:**

*D*—H···*A*	*D*—H	H···*A*	*D*···*A*	*D*—H···*A*
C2*y*—H2*ya*···O2^i^	0.96	2.32	3.177 (10)	148
C3*y*—H3*ya*···O1*y*^ii^	0.96	1.85	2.681 (12)	143

Symmetry codes: (i) −*x*, −*y*, *z*−1/2; (ii) *x*, *y*+1, *z*.
